# Perspectives From the Science-Policy Interface in Animal Health and Welfare

**DOI:** 10.3389/fvets.2019.00382

**Published:** 2019-11-08

**Authors:** Simon J. More

**Affiliations:** UCD Centre for Veterinary Epidemiology and Risk Analysis, University College Dublin, Dublin, Ireland

**Keywords:** animal health, animal welfare, science-policy interface, decision-making, policy, science

## Abstract

The aim of this paper is to present scientific perspectives from the science-policy interface in animal health and welfare, with an emphasis on factors critical to scientific effectiveness. While there is broad acceptance of the value of scientific information to inform policy-making, interactions at the science-policy interface are not without difficulties. The literature highlights the need for scientists to build policy relevance to the research focus from the outset, to engage with policy-makers and other stakeholders throughout, to use platforms to facilitate science-policy dialogue, and to disseminate research findings appropriately. In the author's experience, there are a range of factors linked with effectiveness at the science-policy interface in animal health and welfare including a passion for public interest research, scientific independence, a commitment to scientific quality and openness, the opportunities afforded from partnership and collaboration, and an interest in strategic thinking and systems change. In an increasingly complex and rapidly changing world, an objective evidence base for policy decision-making is more important than ever. There is a need for particular attention to the value of collaboration between the natural and social sciences, a recognition among scientists and policy-makers that science is not value-free, the importance of effective communications, and the need to assess and communicate uncertainty. Further, there are particular challenges with science conducted in support of policy development for industry. It is hoped that this paper will stimulate and contribute to discussion and debate, both among scientists and between scientists and policy-makers, to increase scientific effectiveness at the science-policy interface in animal health and welfare.

## Introduction

Animal health and welfare policies are plans of action; essentially the framework and details that underpins programs in surveillance, control, and eradication, among others. Policy-makers consider a range of factors during decision-making, including available scientific evidence but also social, economic, and political concerns (1, 2). As highlighted by Hueston (2), the policy-making process is influenced by organizational culture and existing rules and regulations, and constrained by legal authorities, political correctness and resource availability.

Many scientists work at the interface between science and policy in animal health and welfare, generating scientific information to inform policy decision-making. At this interface, scientists are seeking both to uphold the integrity of their work and to maximize its value to policy-makers and other stakeholders. Scientists are seeking ‘science-informed policy', where animal health policy is informed by science that is excellent, balanced, and clear.

The aim of this paper is to present scientific perspectives from the science-policy interface, with an emphasis on factors critical to scientific effectiveness, drawing on the literature, and the author's own experiences. The author has worked at the science-policy interface over a number of years, both at a national level in Ireland, as Director of the Centre for Veterinary Epidemiology and Risk Analysis (CVERA) at University College Dublin (UCD) (3) and at the European level, as member and chair of both the Animal Health and Welfare (AHAW) Panel and Scientific Committee of the European Food Safety Authority (EFSA) (4).

## The Science-Policy Interface: an Example

The science-policy interface, essentially the interplay between science and policy, is well-illustrated using the example of climate change, as this concerns players and issues that are recognizable by many in the general population. The ‘science' is primarily represented by the Intergovernmental Panel on Climate Change (IPCC) (5), a United Nations (UN) body established in 1988 and currently with 195 member countries, and the ‘policy' by the so-called Conference of the Parties (COP) to the UN Framework Convention on Climate Change (UNFCCC) (6), who meet formally at the annual UN Climate Change conference. Currently there are 197 Parties to the UNFCCC, including 196 countries as well as the European Union (EU). The role of the IPCC is to provide policymakers with comprehensive scientific assessments (currently in its 6th assessment cycle) on the current state of scientific, technical, and socio-economic knowledge about climate change, its impacts and future risks, and options for reducing the rate at which climate change is taking place. Further, the IPCC periodically releases special reports, most recently on the impact of climate change on the oceans and cryosphere (the frozen parts of the planet) (7). Thousands of experts from relevant scientific disciplines worldwide contribute to the development and multiple reviews of the reports, with the aim to provide the highest standards of scientific excellence, balance, and clarity. Calibrated uncertainty language is used throughout each assessment, to communicate confidence (a qualitative assessment of the validity of each study finding based on the type, amount, quality and consistency of evidence, and the degree of agreement) and likelihood (a quantified measure of uncertainty expressed probabilistically) for each study finding (8). The annual UN Climate Change conference is the global forum for multilateral discussion on matters relating to climate change. In pursuit of this objective, the UNFCCC, also known as the Convention, establishes a framework for decision-making and action-taking, with the objective “to stabilize greenhouse gas concentrations in the atmosphere at a level that would prevent dangerous anthropogenic interference with the climate system” (9). The annual UN Climate Change conference provides the forum for negotiation and compromise toward collective decision-making on the Convention and other legal agreements that were subsequently negotiated, including the Kyoto Protocol in 1997 (establishing legally binding obligations for developed countries to reduce their greenhouse gas emissions) and the Paris Agreement in 2016 (which considered the mitigation of greenhouse gas emissions, adaptation, and finance). Effective interaction between science and policy is critical to international climate negotiations. The international climate regime is built upon a clear understanding of the causes of climate change, and the threats posed by it. Scientific information is also critical to the periodic review of long-term global goals. Science is reliant on the UNFCCC parties to promote and cooperate in research and systematic observation of the climate system (10).

This IPCC-UNFCCC example provides some clarity of the differing roles played by science and policy at the science-policy interface. In the area of animal health and welfare, although the models of engagement may differ, the roles of science and policy at the science-policy interface are surprisingly similar.

## Differing Models of Engagement at the Science-Policy Interface in Animal Health

Models of engagement between scientists and policy-makers in animal health and welfare are likely to vary substantially, depending on a range of factors including the organizational structure, tradition, and the mechanisms used to fund scientific research. The following are examples of science-policy engagement models with which the author is familiar:

- *EFSA in support of the European Commission (EC)*. EFSA is an independent EU agency that conducts scientific assessments in response to requests from the European Commission, the European Parliament and EU Member States. The EFSA's AHAW Panel has produced a series of scientific opinions to support policy decision-making in the EC for African swine fever (ASF) preparedness and response in Europe [for example (11, 12)]. Similarly, the AHAW Panel has developed scientific opinions on animal welfare topics, including the welfare of farmed animals at slaughter (13–16). Although EFSA opinions are developed within a formal, legislated structure (17), there is close contact between the requestor and EFSA from interpretation of the mandate through to the conclusions of the assessment. The opinions conform to relevant in-house guidance documents, including those relating to uncertainty (18).
- *CVERA in support of the Irish Department of Agriculture, Food and the Marine (DAFM)*. Over several decades, CVERA has led research in support of the national bovine tuberculosis (bTB) eradication program in Ireland, seeking to clarify and address constraints to eradication. The national bTB eradication program is managed by DAFM, and the interaction between science and policy has been substantial and ongoing, in identifying research needs, assisting with study design, interpreting study findings, and translating results into policy changes. Research has regularly contributed to policy adjustments, relevant to cattle [including (19, 20)], wildlife (21, 22), and the broader program (23, 24). In the field of animal welfare, CVERA has recently developed, and is currently evaluating, a framework to allow critical evaluation of private animal health and welfare standards in quality assurance programs (25).
- *CVERA in support of Animal Health Ireland (AHI)*. AHI is a public-private partnership, established in 2009 with the aim to contribute to a profitable and sustainable farming and agri-food sector in Ireland through improved animal health (26). Prior to AHI establishment, the initial scientific work (27–30) was undertaken independent of policy, seeking to create an evidence base to underpin discussion with, and consideration by, government and industry policy colleagues. Following AHI establishment, however, there has been a highly interactive partnership between science and policy across a highly applied portfolio of scientific research relating to the eradication of bovine viral diarrhea (BVD) [including (31–33)], the control of Johne's disease (JD) (34–36), and milk quality and intramammary antimicrobial usage (37–40). Policy colleagues contribute substantially to the scientific research, particularly at the start (context setting and question formulation) and at the end of a project (study interpretation and application).

There are other models of engagement at the science-policy interface in animal health and welfare, each influenced by a range of factors including resource availability, and cultural context. Engagement at this interface differs between national and international settings, and in countries at different stages of development. Nonetheless, there is a need to work effectively at the science-policy interface to ensure, as far as possible, that animal health and welfare policy is science-informed.

## Working Effectively at the Science-Policy Interface

### Perspectives From the Literature

There is broad acceptance of the value of scientific information to inform policy-making. This process is facilitated within the EU, where science and policy in animal health are legislatively distinct (17), and each of EFSA's scientific opinions is publicly available. As reasonably suggested by Bogenschneider and Corbett, ‘the pursuit of public good cannot be left solely to the interplay between power and self-interest' (41). Nonetheless, interaction at the science-policy interface is not without difficulties, as has been highlighted in the literature. From the perspective of policy-makers, science can be considered fragmented and uncoordinated, leading to the development of outputs that lack relevance, and usefulness (42). Further, the ‘real world' can be perceived to move more quickly than science can accommodate, with a potential disconnection between what policy-makers want to know, and the answers that science can realistically provide (41). Conversely, and reflecting the different traditions between science and policy, it has been suggested, possibly with some hyperbole, that scientists can view policy as ‘driven by political ideology, conventional wisdom, folklore, and wishful thinking … [representing] the triumph of hope over wisdom, sentiment over demonstrated effectiveness, [and] intuition over evidence' (43).

Broadly, four approaches have been suggested to create an environment for sustained interaction between researchers and policy-makers (42), including:

- *Creating opportunities for interaction*, including through dialogue, mediation, and co-construction of knowledge. It has been suggested that this is achieved more effectively through small groups rather than larger conferences (42).
- *Assembling and synthesizing knowledge and gleaning their policy implications*. This is perhaps most clearly illustrated by the work of the Cochrane Library (44) which seeks to promote evidence-informed health decision-making by producing high-quality, relevant, accessible systematic reviews, and other synthesized research evidence. In veterinary medicine, similar approaches have been used, for example with bovine tuberculosis (45).
- *Improving the way that research is presented, disseminated, and communicated*. Boden et al. (46) outline the different perspectives of scientists and policy-makers, and the importance of ‘knowledge brokers' in the transfer and translation of information between them.
- *Within the scientific community, an improved understanding and appreciation of the nature of political decision-making*. Policy-making operates within an institutional culture that sets powerful constraints on what can and cannot be done (47). It is rational but highly complex, as policy-makers faced many opposing (and often irreconcilable) forces. It is also fluid and unpredictable, influenced by the political process, and error-free decisions are expected to be made with haste. Policy-making favors the status quo (41).

In summary, strategies to advance an evidence-based policy agenda will center on the role of relationships (41). As suggested by Stringer and Dougill (48), it is important for scientists to build policy relevance to the research focus from the outset, to engage with policy-makers and other stakeholders throughout, to use platforms to facilitate science-policy dialogue, and to disseminate research findings appropriately.

### The Author's Perspectives

In the author's experience, there are a range of factors linked with effectiveness at the science-policy interface in animal health and welfare, including a passion for public interest research, scientific independence, a commitment to scientific quality and openness, the opportunities afforded from partnership and collaboration, and an interest in strategic thinking and systems change.

- *Research in the public interest*. At this interface, scientists will be aware of their role in constructively influencing (inter)national policy development in animal health. For this reason, a passion for and commitment to public interest research is an important prerequisite to working effectively in this role.
- *Scientific independence*. Scientific independence must be a key value underpinning scientific contribution at the science-policy interface. Policy-makers have multiple interests to consider during policy development (relating, for example, to governance, social issues, and factors affecting implementation), in addition to science (2). For this reason, it is critical that the scientific evidence provides a robust and factual account of current understanding, unfiltered by those issues that will subsequently be considered in the policy mix. Realistically, therefore, the scientist is seeking to ensure that policy decision-making is science-informed rather than science-led (2). As reflected in the founding regulation of EFSA (17), a key tenet of food safety in the EU is the separation of the processes of risk assessment (science) and risk management (policy decision-making), which was formalized primarily in response to the loss in public confidence in food safety in Europe consequent to the bovine spongiform encephalopathy (BSE) crisis. Similarly, the separation of science (that is, the IPCC) and policy (the UNFCCC) is reflected in the above-mentioned climate change example. Nonetheless, scientific independence has the potential to be one of the most significant challenges for those working at the science-policy interface, in large part as a consequence of the proximity to the politics with which the science is being conducted. Funding also has the potential to impact scientific independence. In the CVERA context, this challenge is being tackled through an enduring commitment to public interest research, to scientific quality and openness through publication, and through partnership and collaborations with other scientific institutions. An independent management board, with policy representation, was recently established to provide independent oversight (49).
- *Scientific quality and openness*. Scientific publication is an essential output of the scientific process providing a benchmark for scientific excellence, a means to promote openness and transparency, and a permanent record for perpetuity. For those working at the science-policy interface, scientific publication also provides scientists with an opportunity to explore and disseminate ideas, including those at odds with the status quo. By definition, scientific knowledge undergoes critique and review and is subject to change (2).
- *Partnership and collaboration* are critical at this interface, both with policy-makers and with other scientists. Scientists must be willing to engage with policy colleagues, to ensure scientific outputs are ‘fit for purpose', which EFSA has described as scientific outputs that are contextual, socially sound and accountable, while remaining scientifically robust (50). Collaborative links between CVERA and other scientific colleagues has offered opportunities for innovation. This is particularly true in the context of methodological advances, for example with modeling [for example (33, 36)] and the social sciences (38, 51).
- *Strategic thinking and systems change*. The scientific process is underpinned by curiosity, comparison and long-term thinking. Given this context, scientists have the potential to contribute valuable strategic perspectives at the science-policy interface. Further to an earlier example, the animal health landscape in Ireland was transformed with the establishment of AHI, which is tackling non-regulatory animal health issues through a process of national dialogue and consensus. In the years prior to AHI establishment, scientists contributed greatly, including through the aforementioned publications (27–30), in support of fundamental change in national approaches to animal health policy.

## Further Reflections

We are facing an increasingly complex and rapidly changing world. Global connectedness has grown rapidly, which has facilitated complex transnational supply chains (52) and increased transboundary movement of people and products (53). Further, human impacts are linked to broader environmental concerns, including climate change (54, 55), species decline (56, 57), and plastics pollution (58, 59). In a recent exploration of possible futures, the Joint Research Center of the European Commission (the EU Science Hub) presented four feasible future global scenarios, each assuming a changing climate (2°C by 2050), progressive natural resource depletion, and an increasing human population (9 billion by 2050) (60). Concurrently, we are in a challenging era when scientific facts are often dismissed or ignored, or where values are increasingly more influential than facts in shaping public opinion (50).

These global changes are entirely relevant to and have important implications for animal health and welfare policies, both internationally and nationally. Critical animal health challenges, such as ASF (61) and antimicrobial resistance (62), are influenced by the same drivers of connectedness and human impacts, among others. These drivers are clearly apparent in the global expansion in ASF, for example, from Georgia in 2007 (63) and subsequently across Eurasia. Animal health and welfare policies also have the potential to positively impact global challenges. For example, disease control/prevention can improve on-farm production efficiencies and can also contribute to the mitigation of greenhouse gases (64).

Given the complexity of these challenges, an objective evidence base for policy decision-making is more important than ever (65), including in animal health and welfare. While there are substantial and ongoing challenges, there is reason to be optimistic. As suggested by Bogenschneider and Corbett (41), ‘empirical evidence and rigorous analysis can play a larger role if we take the time and care to do things right. … the need is there, the interest is there, the science is there'. Nonetheless, there are several areas where particular attention should be paid.

- Policy-makers need knowledge of both the context and the detail with respect to the scientific question, to ensure that they have as complete a picture as possible of the issue at hand. To facilitate this, there is a need for collaboration between the natural and social sciences, to provide policy-makers with an understanding of the ‘why' as well as the ‘what'. Milk quality improvements in Ireland were facilitated by an understanding of both key technical issues (37, 39) and of factors that constrained collective action by stakeholder organizations (38).
- There is a need to recognize that the work of scientists is not value-free (50, 66). Values underpin the decisions that we make, both as people and scientists (1), with the potential to influence at many points during the scientific process, particularly at the start (when choosing the topic of study, when determining the questions to ask, when designing the study to answer these questions) and end (when interpreting the study results, during the framing and communicating of the study findings) (66).
- The importance of effective communication cannot be overstated and has been critical in shifting the views of the Irish farming community with respect to the biosecurity implications of livestock movement (67) and of control measures sufficient to reduce time-to-eradication in both the national BVD (33) and bTB (24) eradication programs.
- There is the need to assess and communicate uncertainty to ensure that scientific conclusions provide reliable information for decision-making. In this context, uncertainty has been defined as all types of limitations in available knowledge that affect the range and probability of possible answers to a particular policy-relevant scientific question (18).

To this point, the discussion has focused on generic challenges at the science-policy interface in animal health and welfare, noting that these are relevant to most situations. When conducting scientific research in support of policy development for or in collaboration with industry (as opposed to government), however, there are several particular (indeed, often additional) challenges that scientists may face. There is a need for a shift in paradigm from ‘certainty' to ‘managed risk' for example, when determining herd JD risk in the national JD control program in Ireland (34, 36). Some consideration will be required on the amount of evidence deemed sufficient for decision-making and subsequent action by industry, somewhat akin to the differing levels of evidence that are sufficient for proof in a civil (‘the balance of probabilities') vs. criminal (‘beyond reasonable doubt') trial (68). Further, non-scientific (often financial) questions frequently predominate, and there is potential for conflict between science and commercial reality.

## Conclusions

This paper focuses on scientific effectiveness at the science-policy interface in animal health and welfare. This issue is increasingly important, given a rapidly changing world and multiple global and local challenges. In this paper, the author draws from the literature and personal experiences, but also from the well-recognized example of climate change. A number of factors are linked to scientific effectiveness at the science-policy interface, including a passion for public interest research, scientific independence, a commitment to scientific quality and openness, the opportunities afforded from partnerships and collaboration, and an interest in strategic thinking and systems change. Despite its importance, there has been little published discussion on this issue in the area of animal health and welfare. It is hoped that this paper will stimulate and contribute to the discussion and debate, both among scientists and between scientists and policy-makers, to increase scientific effectiveness at this interface.

## Author Contributions

SM conceived and wrote the manuscript.

### Conflict of Interest

The author declares that the research was conducted in the absence of any commercial or financial relationships that could be construed as a potential conflict of interest.

## References

[1] Douglas HE Science, Policy and the Value-Free Ideal. Pittsburgh, PA: University of Pittsburgh Press (2009).

[2] Hueston WD. Science, politics and animal health policy: epidemiology in action. Prev Vet Med. (2003) 60:3–12. 10.1016/S0167-5877(03)00078-3 12900145

[3] UCD Centre for Veterinary Epidemiology and Risk Analysis. Available online at: http://www.ucd.ie/cvera/ [accessed July 31, 2019].

[4] European, Food Safety Authority,. Available online at: http://www.efsa.europa.eu [accessed July 31, 2019].

[5] Intergovernmental Panel on Climate Change. Available online at: https://www.ipcc.ch [accessed July 31, 2019].

[6] United, Nations Climate Change,. Available online at: https://unfccc.int [accessed July 29, 2019]

[7] Intergovernmental Panel on Climate Change (IPCC) In: Pörtner H-O, Roberts DC, Masson-Delmotte V, Zhai P, Tignor M, Poloczanska E, Mintenbeck K, Nicolai M, Okem A, Petzold J, Rama B, Weyer N, editors. IPCC Special Report on the Ocean and Cryosphere in a Changing Climate, Summary for Policymakers. Available online at: https://www.ipcc.ch [accessed October 02, 2019].

[8] Mastrandrea MD Mach KJ Plattner G-K Edenhofer O Stocker TF Field CB The IPCC AR5 guidance note on consistent treatment of uncertainties: a common approach across the working groups. Clim Change. (2011) 108:675–91. 10.1007/s10584-011-0178-6

[9] Blobel D Meyer-Ohlendorf N Schlosser-Allera C editors. United Nations Framework Convention on Climate Change. Handbook. Bonn: Climate Change Secretariat (2006).

[10] Understanding the UN climate change regime. United Nations Framework Convention on Climate Change. Available online at: https://unfccc.int/resource/bigpicture/ [accessed July 31, 2019].

[11] More SJ Miranda MA Bicout D Bøtner A Butterworth A Calistri P African swine fever in wild boar. EFSA J. (2018) 16:594 10.2903/j.efsa.2018.5344 PMC7009363 32625980

[12] European Food Safety Authority (EFSA) Boklund A Cay B Depner K Földi Z Guberti V Epidemiological analyses of African swine fever in the European Union (November 2017 until November 2018). EFSA J. (2018) 16:5494 10.2903/j.efsa.2018.5494 PMC7009685 32625771

[13] EFSA Panel on Animal Health and Welfare (AHAW) Scientific Opinion on monitoring procedures at slaughterhouses for bovines. EFSA J. (2013) 11:223 10.2903/j.efsa.2013.3460

[14] EFSA Panel on Animal Health and Welfare (AHAW) Scientific opinion on monitoring procedures at slaughterhouses for sheep and goats. EFSA J. (2013) 11:387 10.2903/j.efsa.2013.3522

[15] EFSA Panel on Animal Health and Welfare (AHAW) Scientific Opinion on monitoring procedures at slaughterhouses for pigs. EFSA J. (2013) 11:13 10.2903/j.efsa.2013.3523

[16] EFSA Panel on Animal Health and Welfare (AHAW) Scientific Opinion on monitoring procedures at slaughterhouses for poultry. EFSA J. (2013) 11:45 10.2903/j.efsa.2013.3521

[17] Regulation (EC) No 178/2002 of the European Parliament and of the Council of 28 January 2002 laying down the general principles and requirements of food law establishing the European Food Safety Authority and laying down procedures in matters of food safety Official Journal L 031. (2002). p. 1–24.

[18] EFSA Scientific Committee Benford D Halldorsson T Jeger MJ Knutsen HK More SJ Guidance on uncertainty analysis in scientific assessments. EFSA J. (2018) 16:54 10.2903/j.efsa.2018.5123 PMC7009727 32625671

[19] Clegg TA Good M Duignan A Doyle R More SJ. Shorter-term risk of *Mycobacterium bovis* in Irish cattle following an inconclusive diagnosis to the single intradermal comparative tuberculin test. Prev Vet Med. (2011) 102:255–64. 10.1016/j.prevetmed.2011.07.014 21855153

[20] Clegg TA Good M Duignan A Doyle R Blake M More SJ. Longer-term risk of *Mycobacterium bovis* in Irish cattle following an inconclusive diagnosis to the single intradermal comparative tuberculin test. Prev Vet Med. (2011) 100:147–54. 10.1016/j.prevetmed.2011.02.015 21474194

[21] Griffin JM Williams DH Kelly GE Clegg TA O'Boyle I Collins JD. The impact of badger removal on the control of tuberculosis in cattle herds in Ireland. Prev Vet Med. (2005) 67:237–66. 10.1016/j.prevetmed.2004.10.009 15748755

[22] Aznar I Frankena K More SJ O'Keeffe J McGrath G De Jong MCM. Quantification of *Mycobacterium bovis* transmission in a badger vaccine field trial. Prev Vet Med. (2018) 149:29–37. 10.1016/j.prevetmed.2017.10.010 29290298

[23] Duignan A Good M More SJ A review of quality control in the national bovine tuberculosis control programme in Ireland. Rev Sci Tech. (2012) 31:845–60. 10.20506/rst.31.3.2166 23520738

[24] More SJ. Can bovine TB be eradicated from the Republic of Ireland? Could this be achieved by 2030? Ir Vet J. (2019) 72:717. 10.1186/s13620-019-0140-x 31057791 PMC6485114

[25] More SJ Hanlon A Marchewka J Boyle LA. Private animal health and welfare standards in quality assurance programmes: a review and proposed framework for critical evaluation. Vet Rec. (2017) 180:612. 10.1136/vr.104107 28465328

[26] Animal, Health Ireland,. Available online at: http://animalhealthireland.ie [accessed July 31, 2019].

[27] More SJ Barrett D The herd health initiative. Ir Vet J. (2005) 58:692–4.

[28] More SJ. Shaping our future: animal health in a global trading environment. Ir Vet J. (2007) 60:540–5. 10.1186/2046-0481-60-9-540 21851699 PMC3113835

[29] More SJ. A case for increased private sector involvement in Ireland's national animal health services. Ir Vet J. (2008) 61:92–100. 10.1186/2046-0481-61-2-92 21851708 PMC3113884

[30] More SJ. Global trends in milk quality: implications for the Irish dairy industry. Ir Vet J. (2009) 62:5–14. 10.1186/2046-0481-62-S4-S5 22081986 PMC3339351

[31] Clegg TA Graham D O'Sullivan P McGrath G More SJ. Temporal trends in the retention of BVD+ calves and associated animal and herd-level risk factors during the compulsory eradication programme in Ireland. Prev Vet Med. (2016) 134:128–38. 10.1016/j.prevetmed.2016.10.010 27836034

[32] Reardon F Graham D Clegg TA Tratalos JA O'Sullivan P More SJ. Potential infection-control benefit of measures to mitigate the risk posed by Trojan dams in the Irish BVD eradication programme. Prev Vet Med. (2018) 157:78–85. 10.1016/j.prevetmed.2018.06.001 30086852

[33] Thulke H-H Lange M Tratalos JA Clegg TA McGrath G O'Grady L. Eradicating BVD, reviewing Irish programme data and model predictions to support prospective decision making. Prev Vet Med. (2018) 150:151–61. 10.1016/j.prevetmed.2017.11.017 29221591

[34] More SJ Sergeant ESG Strain S Cashman W Kenny K Graham D. The effect of alternative testing strategies and bio-exclusion practices on Johne's disease risk in test-negative herds. J Dairy Sci. (2013) 96:1581–90. 10.3168/jds.2012-5918 23295113

[35] Meyer A McAloon CG Tratalos JA More SJ Citer LR Graham D. Modeling of alternative testing strategies to demonstrate freedom from *Mycobacterium avium* ssp. *paratuberculosis* infection in test-negative dairy herds in the Republic of Ireland. J Dairy Sci. (2019) 102:2427–42. 10.3168/jds.2018-14883 30639002

[36] Sergeant ESG McAloon CG Tratalos JA Citer LR Graham D More SJ Evaluation of national surveillance methods for detection of Irish dairy herds infected with *Mycobacterium avium* subspecies *paratuberculosis*. J Dairy Sci. (2019) 102:2525–38. 10.3168/jds.2018-15696 30692009

[37] More SJ Clegg TA O'Grady L. Insights into udder health and intramammary antibiotic usage on Irish dairy farms during 2003–2010. Ir Vet J. (2012) 65:7. 10.1186/2046-0481-65-7 22455802 PMC3376034

[38] Devitt C McKenzie K More SJ Heanue K McCoy F. Opportunities and constraints to improving milk quality in Ireland: enabling change through collective action. J Dairy Sci. (2013) 96:2661–70. 10.3168/jds.2012-6001 23403189

[39] More SJ Clegg TA Lynch PJ O'Grady L. The effect of somatic cell count data adjustment and interpretation, as outlined in European Union legislation, on herd eligibility to supply raw milk for processing of dairy products. J Dairy Sci. (2013) 96:3671–81. 10.3168/jds.2012-6182 23587392

[40] More SJ Clegg TA McCoy F. The use of national-level data to describe trends in intramammary antimicrobial usage on Irish dairy farms from 2003 to 2015. J Dairy Sci. (2017) 100:6400–13. 10.3168/jds.2016-12068 28624279

[41] Bogenschneider K Corbett TJ Evidence-Based Policymaking: Insights From Policy-Minded Researchers and Research-Minded Policymakers. New York, NY: Routledge/Taylor & Francis Group (2011). 10.4324/9780203856390

[42] Saunders L Research and policy: reflections on their relationship. Evid Policy. (2005) 1:383–90. 10.1332/1744264054851568

[43] Davies P What is evidence-based education? Brit J Educ Stud. (1999) 47:108–21. 10.1111/1467-8527.00106

[44] Cochrane Library. Available online at: July 29, 2019.

[45] Godfray HCJ Donnelly CA Kao RR Macdonald DW McDonald RA Petrokofsky G. A restatement of the natural science evidence base relevant to the control of bovine tuberculosis in Great Britain. Proc Biol Sci B. (2013) 280:20131634. 10.1098/rspb.2013.1634 23926157 PMC3757986

[46] Boden L Auty H Goddard P Stott AW Ball N Mellor DJ. Working at the science-policy interface. Vet Rec. (2014) 174:165–7. 10.1136/vr.g1430 24526537

[47] Weiss CH Research-Policy Linkages: How Much Influence Does Social Science Research Have? World Social Science Report. Paris: UNESCO (1999). p. 194–205.

[48] Stringer LC Dougill AJ. Channelling science into policy: enabling best practices from research on land degradation and sustainable land management in dryland Africa. J Environ Manage. (2013) 114:328–335. 10.1016/j.jenvman.2012.10.025 23158525

[49] UCD CVERA Statement of Strategy 2017–20 Centre for Veterinary Epidemiology and Risk Analysis (CVERA). Available online at: http://www.ucd.ie/cvera/t4media/CVERA_StrategicPlan_2017_20.pdf

[50] Devos Y Elliott KC Macdonald P McComas K Parrino L Vrbos D Conducting fit-for-purpose food safety risk assessments. EFSA J. (2019) 17:e170707 10.2903/j.efsa.2019.e170707 PMC7015513 32626444

[51] More SJ McKenzie K Doherty ML Cromie AR Magan MJ. Setting priorities for non-regulatory animal health in Ireland: results from an expert Policy Delphi study and a farmer priority identification survey. Prev Vet Med. (2010) 95:198–207. 10.1016/j.prevetmed.2010.04.011 20554068

[52] Waters D Rinsler S Global Logistics: New Directions in Supply Chain Management. Kogan Page Publishers (2014).

[53] International, Organization for Migration,. Available online at: https://www.iom.int [accessed July 30, 2019].

[54] IPCC Climate Change 2014: Synthesis Report. Contribution of Working Groups I, II and III to the Fifth Assessment Report of the Intergovernmental Panel on Climate Change. Geneva: IPCC (2014). p. 151.

[55] Nolan C Overpeck JT Allen JRM Anderson PM Betancourt JL Binney HA. Past and future global transformation of terrestrial ecosystems under climate change. Science. (2018) 361:920–3. 10.1126/science.aan5360 30166491

[56] Ceballos G Ehrlich PR Dirzo R. Biological annihilation via the ongoing sixth mass extinction signaled by vertebrate population losses and declines. PNAS. (2017) 114:E6089–96. 10.1073/pnas.1704949114 28696295 PMC5544311

[57] Sánchez-Bayo F Wyckhuys KAG Worldwide decline of the entomofauna: a review of its drivers. Biol Conserv. (2019) 232:8–27. 10.1016/j.biocon.2019.01.020

[58] Jamieson AJ Brooks LSR Reid WDK Piertney SB Narayanaswamy BE Linley TD. Microplastics and synthetic particles ingested by deep-sea amphipods in six of the deepest marine ecosystems on Earth. Royal Soc Open Sci. (2019) 6:180667. 10.1098/rsos.180667 30891254 PMC6408374

[59] Lebreton L Slat B Ferrari F Sainte-Rose B Aitken J Marthouse R. Evidence that the Great Pacific Garbage Patch is rapidly accumulating plastic. Sci Rep UK. (2018) 8:4666. 10.1038/s41598-018-22939-w 29568057 PMC5864935

[60] Mylona K Maragkoudakis P Bock A-K Wollgast J Caldeira S Ulberth F Delivering on EU Food Safety and Nutrition in 2050 – Future Challenges and Policy Preparedness. EUR27957 EN. Luxembourg: Publications Office of the European Union (2016).

[61] Sánchez-Cordón PJ Montoya M Reis AL Dixon LK. African swine fever: a re-emerging viral disease threatening the global pig industry. Vet J. (2018) 233:41–8. 10.1016/j.tvjl.2017.12.025 29486878 PMC5844645

[62] Woolhouse MEJ Ward M van Bunnik B Farrar J. Antimicrobial resistance in humans, livestock and the wider environment. Philos Trans R Soc Lond B Biol Sci. (2015) 370:20140083. 10.1098/rstb.2014.0083 25918441 PMC4424433

[63] Rowlands RJ Michaud V Heath L Hutchings G Oura C Vosloo W. African swine fever virus isolate, Georgia, 2007. Emerging Infect Dis. (2008) 14:1870–4. 10.3201/eid1412.080591 19046509 PMC2634662

[64] Williams A Chatterton J Hateley G Curwen A Elliott J A systems-life cycle assessment approach to modelling the impact of improvements in cattle health on greenhouse gas emissions. Adv Anim Biosci. (2015) 6:29–31. 10.1017/S2040470014000478

[65] SAPEA Science Advice for Policy by European Academics Making Sense of Science for Policy Under Conditions of Complexity and Uncertainty. Berlin: SAPIA (2019).

[66] Elliott KC Managing value-laden judgements in regulatory science and risk assessment. EFSA J. (2019) 17:e170709 10.2903/j.efsa.2019.e170709 PMC7015493 32626446

[67] McGrath G Tratalos JA More SJ. A visual representation of cattle movement in Ireland during 2016. Ir Vet J. (2018) 71:18. 10.1186/s13620-018-0129-x 30202515 PMC6128986

[68] Weiss C Expressing scientific uncertainty. Law Probab Risk. (2003) 2:25–46. 10.1093/lpr/2.1.25

